# Conspiracy mentality among informal caregivers as a risk factor for caregiver burden, mental health, perceived loneliness and social isolation during the COVID-19 pandemic: findings of a representative online study from Germany

**DOI:** 10.1007/s11136-022-03177-0

**Published:** 2022-07-05

**Authors:** Larissa Zwar, Hans-Helmut König, André Hajek

**Affiliations:** Department of Health Economics and Health Services Research, University Medical Center Hamburg-Eppendorf, Martinistraße 52, 20246 Hamburg, Germany

**Keywords:** Informal care, Conspiracy mentality, Mental health, Loneliness, Social isolation, Burden

## Abstract

**Purpose:**

This study aims to analyze if and how conspiracy mentality is associated with mental health, burden and perceived social isolation and loneliness of informal caregivers of older individuals with care needs.

**Methods:**

A quantitative, cross-sectional study was conducted. Participants had to be at least 40 years of age and were drawn randomly from the German online panel forsa.omninet and questioned between the 4th and 19th of March 2021. A sample of 489 informal caregivers (relatives and non-relatives supporting individuals aged ≥ 60 years) was questioned. Conspiracy mentality, depressive symptoms, loneliness and social exclusion were measured with validated instruments (e.g., The Conspiracy Mentality Questionnaire). Questions referred to the last three months prior to assessment. Multiple linear regression analyses, adjusted for sociodemographic, economic and health factors and indicators of the pandemic, were conducted.

**Results:**

Findings indicate a significant positive association between conspiracy mentality and caregiver burden, loneliness, social exclusion, and depressive symptoms. No gender differences were found for any outcome.

**Conclusions:**

The results indicate that conspiracy mentality could be a risk factor for mental health, perceived social isolation and loneliness, and contribute to increased caregiver burden among informal caregivers of older care recipients during the COVID-19 pandemic. Accordingly, informal caregivers could benefit from actions focused on reducing conspiracy mentality during a health crisis, which could improve psychosocial health and wellbeing in this vulnerable group.

**Supplementary Information:**

The online version contains supplementary material available at 10.1007/s11136-022-03177-0.

## Plain English summary

Providing care for older adults (60 years and older, e.g., health-impaired partners or parents) is a challenging task and can result in reduced health and wellbeing for informal caregivers. To enable adequate support to be provided to these caregivers, it is necessary to know which factors may either increase the risk or provide a buffer against poorer health and wellbeing—in particular during the challenging time of the COVID-19 pandemic. We assume that the inclination to believe in conspiracy theories during the COVID-19 pandemic could either worsen or improve the stress level of informal caregivers and its consequences for health and wellbeing. Thus, we analyzed if and how the inclination to believe in conspiracy theories is associated with the perceived social integration, mental health and burden of caregiving, during the pandemic. It was shown that a higher inclination to believe in conspiracy theories during the COVID-19 pandemic was associated with worse mental health, increased social isolation and loneliness, and contributed to worse caregiver burden among informal caregivers. Thus, conspiracy beliefs should be given more attention in particularly vulnerable groups, such as informal caregivers. Also, informal caregivers could benefit from actions focused on reducing conspiracy mentality during the COVID-19 pandemic, which could help to improve their psychosocial health and wellbeing.

## Introduction

The COVID-19 pandemic has been officially declared in March 2020, after having started at the end of 2019 with the first occurrence of the SARS-CoV-2 virus [1]. It led to changes in every area of life, including changes in work life (e.g., home office), family life (e.g., temporary or long-term closure of care and educational facilities), social life (e.g., restrictions and new rules for social contacts, such as new hygiene and distance concepts) and leisure activities (e.g., closure of leisure facilities, such as cinemas, theaters or fitness centers) [e.g., 2–4]. These restrictions and changes in work and private life forced individuals to adapt their daily life quickly and be prepared for further changes due to the uncertainty of the pandemic development. This can cause stress as illustrated in the transactional stress-process model from Lazarus [5]. According to the model, stress results from the evaluation of a (new or changed) situation as threatening or damaging and the evaluation of one’s own resources as insufficient for managing this situation. Thus, the pandemic is a potentially stressful situation or stressor, which can result in negative psychosocial and health consequences [6, 7].

Some individuals are expected to be particularly endangered by the pandemic’s potential for stress. Among these are *informal caregivers* of older people, i.e., individuals who provide care for adult relatives or friends with health- or age-related impairments and care needs. In Germany, informal caregiving is a central aspect of the health care system. Individuals can apply for benefits from nursing insurance, which are based on their level of care needs [8]. About 70% of the community-dwelling individuals with registered care needs are cared for solely by informal caregivers [9]. Pearlin et al. [10] formulated a stress-process model focused only on the informal caregiving context. The model illustrates the factors which can influence the stress process and its consequences for health and wellbeing of the informal caregivers. These are context and background factors, such as the sociodemographic background of the caregiver, and primary and secondary stressors, such as care intensity and role conflicts of the caregiver. The pandemic changed many of these factors. For example, pandemic countermeasures restricted access or availability of care and educational facilities for children and professional care options for older care recipients and included changes in work life as well (e.g., home office) [e.g., 2–4]. Unsurprisingly, more role conflicts between family and work life, and increased caregiving time and intensity were found [11, 12]. Thus, the COVID-19 pandemic is a context factor which adds further challenges to the already stressful caregiving situation and can thereby influence its consequences for health and wellbeing. Some evidence for this has already been found in terms of increased caregiver burden during home confinement [13] and worse mental health [14] compared to non-caregivers during the pandemic. To be able to provide adequate support, research on *risk and resiliency factors* for informal caregiver’s health and wellbeing, which occur in particular during the COVID-19 pandemic, is needed. We assume that one such factor could be the *belief in conspiracy theories*.

*Conspiracy theories* are theories that locate the cause for a major event in a secret plot of a powerful group [15, 16]. Previous findings indicate that individuals who believe in one conspiracy theory usually believe in others as well, even if they are in contradiction to each other [15, 17]. This inclination to believe in conspiracy theories is called *conspiracy mentality* [18]. During the COVID-19 pandemic, there has been an upsurge of conspiracy theories to explain either the existence (e.g., the virus is a hoax) or the origin (e.g., the virus was created in a laboratory for malicious reasons by a specific country) of the SARS-CoV-2 virus [19]. Further conspiracy theories focus on the pandemic countermeasures (e.g., microchips in vaccine to control the population) [19].

In research on conspiracy beliefs, three motives have been identified among individuals believing in conspiracy theories[20]: *Epistemic motives* refer to the need for having an explanation for an event, such as the COVID-19 pandemic, and can be motivated by the need to reduce uncertainty or to find meaning in an event. *Existential motives* are based on the need to feel safe and have control over one’s context; feeling powerless or threatened are therefore existential motives for turning to conspiracy theories. Last, *social motives* are based on the need to belong to a group and to preserve a positive image of oneself, such as feeling unique. These motives are expected to underlie informal caregivers’ beliefs in conspiracy theories during the COVID-19 pandemic as well. As described before, the caregiving situation can already be stressful under regular circumstances and this is worsened by the pandemic situation. Being primarily responsible for the high risk group of older individuals (with preexisting illnesses) [9, 21], while often being part of this high risk group themselves (many caregivers are aged 60 years and older; [22]) places informal caregivers in a particularly threatened but also difficult situation, since they often had to decide on their own how best to proceed with caregiving. Feelings of uncertainty, worrying and wondering how to handle the caregiving situation during the pandemic, as well as perceiving themselves and their care recipients to be particularly endangered, have been reported [12, 23–25]. During the time of data assessment of this study, the vaccinations had slowly started but no COVID-19-specific medication was available yet. Also, pandemic measures had been changed and extended repeatedly and irregularly in dependence on the pandemic situation [e.g., 2–4], which can contribute to further uncertainty and confusion. Moreover, reports of loneliness among the general population [6] and lower social participation among caregivers [14], indicate that perceived social integration was threatened during the pandemic as well. Thus, all three motives, epidemic, existential and social motives, can be expected to be prevalent among informal caregivers during the COVID-19 pandemic and to motivate their readiness to believe in conspiracy theories. This raises the question if and how believing in conspiracy mentality is associated with informal caregivers psychosocial wellbeing and level of burden.

*Burden* refers to the perceived psychosocial stressful aspects of care provision and is therefore a good indicator of the stress level caused by informal caregiving [26] and an important determinant of mental health [27, 28] and quality of life [29]. *Social isolation* and *loneliness* are both important determinants of morbidity and survival [30–32]. Also, *mental health* is often worse among informal caregivers than among non-caregivers [33, 34], even during the pandemic [14, 35]. Thus, analyzing if conspiracy mentality is a *risk or resilience factor* for these outcomes among informal caregivers, particularly during such a difficult time as the COVID-19 pandemic, would extend our knowledge on the informal caregiving situation and could help to inform future interventions. As explained before, various factors can be of relevance for the stress process in informal caregivers and influence these health and wellbeing outcomes [5, 10]. The level of *conspiracy mentality* could be such a factor that affects the stress process by influencing the evaluation of the manageability of the caregiving situation during the pandemic. Two hypotheses will be explored: (1) If conspiracy mentality fulfills the epistemic, existential and social needs, informal caregivers with higher levels of conspiracy mentality would perceive their situation as more manageable. In consequence, burden and depressive symptoms as well as perceived social isolation and loneliness should be lower. (2) However, if conspiracy mentality does not fulfill these needs, higher levels of conspiracy mentality would instead worsen informal caregiver’s perception of the manageability of the caregiving situation. In this case, their psychosocial wellbeing and level of burden should worsen.

So far, informal caregivers have not been investigated regarding their conspiracy mentality or its association with their wellbeing and health. Yet, other research already indicates a detrimental effect of conspiracy mentality on wellbeing and health. For example, conspiracy beliefs predicted worse mental health in terms of distress and anxiety disorder as well as lower life and job satisfaction among healthcare workers [36]. Also, there are previous findings suggesting that believing in conspiracy theories does not necessarily lead to the fulfillment of the three motives or needs listed above. For example, increased feelings of powerlessness [16, 20, 37] and (fear of) more social isolation [38, 39] have been reported.

### Aim

Thus, in this study we aimed to explore *if* and *how* conspiracy mentality is associated with burden, mental health (in terms of depressive symptoms), and perceived loneliness and social exclusion among informal caregivers of older individuals (aged ≥ 60 years) during the COVID-19 pandemic. Findings could provide first indications if conspiracy mentality should be taken into consideration as a factor of resilience, as indicated by improving the outcomes, or as a risk factor, as indicated by worsening the outcomes.

## Methods

### Sample

Participants were randomly sampled from the online panel forsa.omninet in cooperation with the market research institute forsa. Forsa.omninet is a population-based Online panel. It is based on the forsa.omnitel panel which is drawn randomly according to the ADM-phone sampling scheme and recruited via phone. We included only adults aged 40 years and older, because previous findings have shown that the majority of caregivers is in the second half of life [22, 40]. In total, 3022 participants were questioned. Data assessment took place from the 4th to the 19th of March 2021. In this study, we focus only on informal caregivers, therefore the analytical sample of this study consisted solely of the 489 participants, who reported to provide informal care. Informal care referred to providing care for someone aged ≥ 60 years (i.e., support for relatives, friends or others) with care needs in terms of support with, for example, household tasks or personal hygiene at least once a week during the first winter of the COVID-19 pandemic (between December 2020 and March 2021). We focused on caregiving for adults aged ≥ 60 years since these care recipients are part of the group with a high risk for a worse development and higher mortality risk due to COVID-19 [41]. The non-caregiving participants were not analyzed in this study. We expect the sample size to be adequate for the adjusted regression analyses, based on our a priori analysis to identify the sample size needed to achieve a power between 0.60 and 0.95 when including 23 predictors, and expecting a medium effect based on *α* = 0.05. The test indicated a sample size between 120 and 230 is needed, our sample of *N* = 489 is therefore expected to be sufficient. The questionnaire and all information on the study was presented in German.

All participants provided written informed consent and an ethics approval was provided by the Ethics Committee of the Center for Psychosocial Medicine of the University Medical Center Hamburg-Eppendorf (LPEK-0239).

### Variables

#### Dependent variables

*Caregiver burden* was measured with the Burden Scale for Family Caregivers short version (BSFC-s) [26]. The scale is based on the Transactional Stress Model from Lazarus and Folkman [5] and the caregiving-specific stress-process model from Pearlin et al. [10] and refers to the psychosocial stress of caregiving as appraised by the caregiver. The scale measures burden of informal caregiving with 10 items (e.g., “I often feel physically exhausted.”), which are summed up to a sum score (Range 0–30). Items were recoded so higher scores indicate higher burden. It is a well-established instrument which had good reliability in this study (Cronbach’s *α* = 0.94).

*Loneliness* was measured with the De Jong Gierveld Loneliness Short Scale [42, 43]. It expresses the lack of qualitatively good relationships (e.g., “I miss people who make me feel comfortable.”). The mean score based on 6 items ranges from 1 to 4. Some items were recoded so that higher scores indicate higher loneliness. Good reliability of the instrument has been shown in this study (Cronbach’s *α* = 0.73).

*Social exclusion* was measured with the scale from Bude and Lantermann [44]. It differs from loneliness by measuring the level of perceived exclusion from society (e.g., “I have the feeling that I don't really belong to society at all.”). The scale includes 6 items based on which a mean score is built (Range 1–4). Higher scores indicate higher levels of perceived social exclusion and Cronbach’s *α* in this study was 0.91.

*Depressive symptoms* were measured with the Patient Health Questionnaire-9 (PHQ-9) [45, 46]. The instrument consists of 9 items asking how often participants had felt impaired by specific complaints during the last weeks (e.g., “little interest or pleasure in one’s activities”), which are summed up to a score (Range 0–27), with higher scores indicating more depressive symptoms. The questionnaire had a Cronbach’s *α* of 0.87 in this study.

#### Independent variables

*Conspiracy mentality* was measured with the German version of the 5-item-scale from Bruder et al. [18], called Conspiracy Mentality Questionnaire (CMQ). A sum score is calculated based on the items which assess the general susceptibility to believe in conspiracy theories (Range 0–50). Higher scores indicate higher conspiracy mentality. The instrument has been validated and had good test–retest reliability in previous research (*r* = 0.84) [18] and a Cronbach’s *α* of 0.87 in this study.

*Caregiving time* was measured by asking individuals to report the number of hours per week during which they provide care for an individual aged 60 years or older, such as, support with household tasks or personal care or supervision (Range 0–168 h/week). *Self-rated health* was measured with a single question asking participants to evaluate their current general health status (Range 1–5). Higher scores indicate better health. *Social support* by family and friends was measured with the 6-item short version of the Lubben Social Network Scale (LSNS-6; [47]. The general sum score ranges from 0 to 30, with higher scores indicating higher support levels. *Perceived danger* by the pandemic for *oneself (caregivers)* and for their *care recipient* were both measured with a single item designed by our research group for this project (‘How much do you feel threatened by the pandemic?’, ‘How much of a risk do you think the COVID-19 pandemic poses to the person you primarily care for?’). The mean score ranged from 1 to 5 (Range 1–5); higher scores indicate higher perceived danger. A pretest confirmed face validity of the questions. Information on the *sociodemographic* and *socioeconomic background* was collected as well. This included *age* (40 years or older), *gender* [male, female, diverse (if they cannot be categorized into the male or female gender due to a variation in their sex development [48])], *marital status* (married, divorced, widowed, single), *employment status* during the pandemic phase that we assessed (employed full-time, employed part-time, marginally employed, retired, unemployed), *highest educational degree* (no school qualification, lower secondary school, intermediate secondary school, polytechnic secondary school, qualification for applied upper secondary school, upper secondary school), *living situation* (living alone in private home, living together with others in private home, living in assisted living/nursing care home/retirement home), and having *children in one’s household* (none; yes, aged younger than 14 years; yes, aged between 14 and 18 years).

### Statistics

Descriptive statistics are given for the complete sample. To analyze the research question, multiple linear regression analyses were conducted. Covariates were chosen based on theoretical consideration and previous research [10, 16] in order to prevent biased results due to unobserved variables. These covariates included sociodemographic background and the caregiver’s self-rated health, caregiving time and social support as well as the level of perceived danger for themselves and for their care recipients. All models were adjusted for all of these covariates. Robust standard errors were calculated for all models. Gender-sensitive analyses were conducted in addition. While previous findings are mixed regarding gender differences in conspiracy mentality [16, 49, 50], they indicate gender differences in burden, mental health and loneliness among informal caregivers [34, 51, 52]. Thus, analyses with gender as moderator were conducted. All models which we conducted are reported in the results section and depicted in Tables 2 and 3, while Table 1 provides a description of the sample.

**Table 1:** Descriptive statistics for the sample of informal caregivers (*N* = 489)

	*N* (%)/M(SD)
Gender (%)	
Male	188 (38.45)
Female	301 (61.55)
Age	58.19 (9.66)
Highest educational degree (%)	
No school qualification	–
Lower secondary school	91 (18.61)
Intermediate secondary school	173 (35.38)
Polytechnic secondary school	40 (8.18)
Qualification for applied upper secondary school	53 (10.84)
Upper secondary school	126 (25.77)
Marital status (%)	
Married	345 (70.55)
Divorced	54 (11.04)
Widowed	24 (4.91)
Single	64 (13.09)
Employment status (%)	
Employed (full-time)	189 (38.65)
Employed (part-time)	104 (21.27)
Marginally employed	19 (3.89)
Retired	128 (26.18)
Unemployed	47 (9.61)
Living situation (%)	
Living alone in private household	114 (23.31)
Living together with other ins private household	368 (75.26)
Living in assisted living/nursing care home/retirement home	3 (0.61)
Children in one’s household (%)	
None	385 (78.73)
Yes, younger than 14 years	43 (8.79)
Yes, between 14 and 18 years	56 (11.45)
Caregiving time (h/week)	12.20 (19.57)
Self-rated health	3.43 (.90)
Social support	15.74 (4.88)
Perceived danger for caregiver	2.85 (.98)
Perceived danger for care recipient	3.21 (1.16)
Conspiracy mentality	19.67 (10.97)
Social exclusion	1.45 (.65)
Loneliness	2.00 (.63)
Burden	8.49 (7.62)
Depressive symptoms	5.47 (4.76)

*Conspiracy mentality* (CMQ, Range 0–50), higher scores indicate higher conspiracy mentality; *burden* (BSFC-s, Range 0–30), higher scores indicate higher caregiver burden; *loneliness* (de Jong Gierveld Scale, Range 1–4), higher scores indicate higher loneliness; *social exclusion* (Bude & Lantermann scale, Range 1–4), higher scores indicate higher social exclusion; *depressive symptoms* (PHQ-9, Range 0–27), higher scores indicate more depressive symptoms; *self-rated health* (Range 1–5), higher scores indicate better health; *social support* (Lubben’s social network scale, Range 0–30), higher scores indicate more social support; *perceived danger for oneself* (caregiver) *and for care recipient* (Range 1–5), higher scores indicate higher levels of perceived danger

Missing values (see Online Appendix, Table A1) were mostly below 5%, with the exception of caregiving time. Of the participants, 10.63% reported not knowing their caregiving time, while 2.66% provided no information without naming a reason. In the multiple regression analyses listwise deletion was used for missing values. This method drops all individuals with missing values in at least one variable and can be used if values are missing completely at random (MCAR). However, relevant information may have been excluded. Therefore, we additionally conducted structural equation analyses using full information maximum likelihood (FIML) estimation to check our results. FIML can be used when MCAR or missing at random (MAR) applies [53]. It uses all available information of the data and often results in less biased and more efficient estimates than listwise deletion [53]. Robust standard errors were calculated for all analyses. The alpha level was set at 5% and all analyses were conducted with Stata version 16.1 (Stata Corp., College Station Texas) and sample size calculations with G-Power version 3.1.9.7 [54].

## Results

### Descriptive results

The descriptive results are provided in detail in Table 1. The sample was on average 58.19 (SD = 9.66) years of age (Range 41–95) and the majority was female (61.55%). None of the caregivers categorized themselves as diverse in the gender variable. Conspiracy mentality was on average moderate (*M* = 19.67, SD = 10.97). Regarding the outcomes, social exclusion (*M* = 1.45, SD = 0.65) and loneliness (*M* = 2.00, SD = 0.63) were perceived to be low to moderate. Burden was on average on a lower level (*M* = 8.49, SD = 7.62) and mild depressive symptoms were reported (*M* = 5.47, SD = 4.76).

### Results of multiple regression analyses

The adjusted regression analyses for burden indicate a significant association between conspiracy mentality and burden (*b* = 0.10, *p* < 0.01, CI [0.03; 0.18], *R*^2^ = 0.21). A significant association was also found between conspiracy mentality and loneliness (*b* = 0.01, *p* < 0.01, CI [0.00; 0.01]; *R*^2^ = 0.31), and between conspiracy mentality and social exclusion (*b* = 0.02, *p* < 0.001, CI [0.01; 0.02]; *R*^2^ = 0.35). A significant association was found between conspiracy mentality and depressive symptoms (*b* = 0.10, *p* < 0.001, CI [0.06; 0.14], *R*^2^ = 0.33) as well. For further results, see Table 2.

**Table 2:** Results of the adjusted regression analyses for the outcomes burden, loneliness, social exclusion and depressive symptoms

	(1)	(2)	(3)	(4)
	Burden	Loneliness	Social exclusion	Depressive symptoms
Conspiracy mentality	0.10**	0.01**	0.02***	0.10***
	(0.04)	(0.00)	(0.00)	(0.02)
Caregiving time	0.05 +	− 0.00 +	0.00	0.00
	(0.03)	(0.00)	(0.00)	(0.01)
Social support	− 0.37***	− 0.05***	− 0.02***	− 0.16***
	(0.08)	(0.01)	(0.01)	(0.04)
Gender (ref. male)	1.41 +	0.03	0.09	0.75
	(0.82)	(0.06)	(0.06)	(0.47)
Age	− 0.05	− 0.01	− 0.01	− 0.13***
	(0.06)	(0.00)	(0.00)	(0.04)
Marital status (ref. Married)				
Divorced	− 1.92	− 0.01	0.15	− 0.13
	(1.28)	(0.08)	(0.10)	(0.75)
Widowed	− 2.66	0.28 +	0.02	1.09
	(2.25)	(0.15)	(0.09)	(0.84)
Single	− 2.03 +	0.13	0.22*	− 0.13
	(1.22)	(0.11)	(0.11)	(0.81)
Highest educational degree (ref. upper secondary school)				
Lower secondary school	− 0.63	− 0.04	− 0.01	− 0.76
	(1.12)	(0.09)	(0.08)	(0.64)
Intermediate secondary school	1.31	− 0.02	0.09	− 0.08
	(0.95)	(0.07)	(0.07)	(0.57)
Polytechnic secondary school	0.02	− 0.06	− 0.01	− 0.98
	(1.53)	(0.11)	(0.09)	(0.82)
Qualification for applied upper secondary school	0.49	0.17 +	0.18 +	0.41
	(1.34)	(0.10)	(0.10)	(0.81)
Employment status (ref. employed (full-time))				
Employed (part-time)	− 0.27	0.00	0.00	− 0.57
	(1.04)	(0.08)	(0.08)	(0.54)
Marginally employed	− 3.99*	− 0.19	− 0.23 +	− 0.72
	(1.86)	(0.14)	(0.14)	(0.76)
Retired	− 1.14	− 0.17 +	− 0.03	− 0.02
	(1.25)	(0.09)	(0.09)	(0.75)
Unemployed	− 0.74	0.00	0.29*	1.86*
	(1.64)	(0.11)	(0.13)	(0.92)
Living situation (ref. living alone in private household)				
Living together with other ins private household	− 1.07	− 0.01	− 0.08	0.03
	(1.27)	(0.09)	(0.08)	(0.69)
Living in assisted living/nursing care home/retirement home	− 5.81*	− 0.26 +	− 0.65**	− 1.04
	(2.56)	(0.14)	(0.22)	(1.58)
Children in one’s household (ref. None)				
Yes, younger than 14 years	− 2.06 +	0.02	0.19	− 1.03
	(1.23)	(0.11)	(0.12)	(0.72)
Yes, between 14 and 18 years	0.46	0.00	− 0.06	− 0.01
	(1.10)	(0.09)	(0.07)	(0.69)
Self-rated health	− 1.31**	− 0.14***	− 0.14***	− 1.87***
	(0.50)	(0.03)	(0.03)	(0.29)
Perceived danger for caregiver (oneself)	0.76 +	0.06 +	0.08*	0.19
	(0.44)	(0.03)	(0.03)	(0.28)
Perceived danger for care recipient	0.17	− 0.00	0.04 +	0.39 +
	(0.34)	(0.03)	(0.03)	(0.21)
Constant	17.41***	3.29***	1.95***	18.09***
	(4.83)	(0.35)	(0.35)	(2.90)
Observations	372	385	373	375
*R*^2^	0.21	0.31	0.35	0.33

OLS regression analyses were conducted; unstandardized regression coefficients are given and robust standard errors in parentheses. *Conspiracy mentality* (CMQ, Range 0–50); *burden* (BSFC-s, Range 0–30); *loneliness* (de Jong Gierveld Scale, Range 1–4); *social exclusion* (Bude & Lantermann scale, Range 1–4); *depressive symptoms* (PHQ-9, Range 0–27); *self-rated health* (Range 1–5); *social support* (Lubben’s social network scale, Range 0–30); *perceived danger for oneself* (caregiver) *and for care recipient* (Range 1–5)

Level of significance: ***p < 0.001, **p < 0.01, *p < 0.05, ^+^*p* < 0.10

### Results of moderator analyses

We tested gender differences by including gender as a moderator in the four models (Table 3). No significant interaction effects were found for any of the outcomes (model 1 burden: *b* = 0.01, *p* = 0.842, CI [− 0.12; 0.15]; model 2 loneliness: *b* = − 0.00, *p* = 0.628, CI [− 0.01; 0.01]; model 3 social exclusion: *b* = − 0.01, *p* = 0.227, CI [− 0.01, 0.00]; model 4 depressive symptoms: *b* = − 0.05, *p* = 0.206, CI [− 0.14; 0.03]).

**Table 3:** Additional adjusted regression analyses with gender as moderator

	(1)	(2)	(3)	(4)
	Burden	Loneliness	Social exclusion	Depressive symptoms
Conspiracy mentality	0.09*	0.01*	0.02***	0.13***
	(0.05)	(0.00)	(0.00)	(0.03)
Gender (ref. male)	1.15	0.08	0.21*	1.72*
	(1.46)	(0.12)	(0.10)	(0.82)
Conspiracy mentality × gender	0.01	− 0.00	− 0.01	− 0.05
	(0.07)	(0.01)	(0.01)	(0.04)
Caregiving time	0.05 +	− 0.00 +	0.00	0.00
	(0.03)	(0.00)	(0.00)	(0.01)
Social support	− 0.37***	− 0.05***	− 0.03***	− 0.17***
	(0.08)	(0.01)	(0.01)	(0.04)
Age	− 0.05	− 0.01	− 0.01	− 0.13***
	(0.06)	(0.00)	(0.00)	(0.04)
Marital status (ref. Married)				
Divorced	− 1.94	− 0.01	0.16	− 0.08
	(1.29)	(0.08)	(0.10)	(0.75)
Widowed	− 2.66	0.28 +	0.03	1.11
	(2.25)	(0.15)	(0.09)	(0.85)
Single	− 2.03 +	0.13	0.22*	− 0.12
	(1.23)	(0.11)	(0.11)	(0.81)
Highest educational degree (ref. upper secondary school)				
Lower secondary school	− 0.63	− 0.04	− 0.01	− 0.77
	(1.12)	(0.09)	(0.08)	(0.64)
Intermediate secondary school	1.30	− 0.02	0.09	− 0.06
	(0.95)	(0.07)	(0.07)	(0.57)
Polytechnic secondary school	0.02	− 0.06	− 0.01	− 0.98
	(1.53)	(0.11)	(0.10)	(0.83)
Qualification for applied upper secondary school	0.50	0.17	0.17 +	0.36
	(1.34)	(0.10)	(0.10)	(0.81)
Employment status [ref. employed (full-time)]				
Employed (part-time)	− 0.28	0.00	0.01	− 0.53
	(1.04)	(0.08)	(0.08)	(0.54)
Marginally employed	− 4.01*	− 0.19	− 0.22 +	− 0.67
	(1.86)	(0.14)	(0.14)	(0.79)
Retired	− 1.14	− 0.18 +	− 0.03	− 0.05
	(1.25)	(0.09)	(0.09)	(0.75)
Unemployed	− 0.73	0.00	0.29*	1.85*
	(1.64)	(0.11)	(0.13)	(0.92)
Living situation (ref. living alone in private household)				
Living together with other ins private household	− 1.06	− 0.01	− 0.08	0.02
	(1.27)	(0.09)	(0.08)	(0.69)
Living in assisted living/nursing care home/retirement home	− 5.89*	− 0.24	− 0.60**	− 0.67
	(2.67)	(0.16)	(0.21)	(1.49)
Children in one’s household (ref. None)				
Yes, younger than 14 years	− 2.07 +	0.02	0.19	− 1.01
	(1.24)	(0.11)	(0.12)	(0.72)
Yes, between 14 and 18 years	0.47	0.00	− 0.06	− 0.04
	(1.11)	(0.09)	(0.07)	(0.69)
Self-rated health	− 1.30*	− 0.14***	− 0.15***	− 1.91***
	(0.50)	(0.03)	(0.04)	(0.29)
Perceived danger for caregiver (oneself)	0.76 +	0.06 +	0.08*	0.20
	(0.44)	(0.03)	(0.03)	(0.29)
Perceived danger for care recipient	0.17	− 0.00	0.04 +	0.39 +
	(0.34)	(0.03)	(0.03)	(0.21)
Constant	17.54***	3.27***	1.90***	17.61***
	(4.88)	(0.35)	(0.34)	(2.85)
Observations	372	385	373	375
*R*^2^	0.21	0.31	0.36	0.34

OLS regression analyses were conducted; unstandardized regression coefficients are given and robust standard errors in parentheses. *Conspiracy mentality* (CMQ, Range 0–50); *burden* (BSFC-s, Range 0–30); *loneliness* (de Jong Gierveld Scale, Range 1–4); *social exclusion* (Bude & Lantermann scale, Range 1–4); *depressive symptoms* (PHQ-9, Range 0–27); *self-rated health* (Range 1–5); *social support* (Lubben’s social network scale, Range 0–30); *perceived danger for oneself* (caregiver) *and for care recipient* (Range 1–5)

Level of significance: ***p < 0.001, **p < 0.01, *p < 0.05, ^+^*p* < 0.10

### Results of structural equation analyses

In additional analyses, using structural equation analysis with FIML (see Online Appendix, Table A2), conspiracy mentality was still significantly associated with burden (*b* = 0.10, *p* < 0.01, CI [0.04, 0.18]), loneliness (*b* = 0.01, *p* < 0.001, CI [0.00, 0.013]), social exclusion (*b* = 0.02, *p* < 0.001, CI [0.01, 0.02]) and depressive symptoms (*b* = 0.10, *p* < 0.001, CI [0.06; 0.13]).

## Discussion

This study is the first to analyze the research questions if and how conspiracy mentality of informal caregivers of individuals in old age is associated with their perceived burden of caregiving, their mental health and their perceived social isolation and loneliness during the COVID-19 pandemic. In brief, the findings indicate that conspiracy mentality was associated with a worsening of all outcomes. Our findings add to previous research on negative consequences for health and wellbeing of conspiracy mentality that had been found for professional caregivers, i.e., health care workers [36].

We had assumed that the conspiracy mentality of informal caregivers could influence their evaluation of the manageability of the caregiving situation during the pandemic and through this would influence the health, burden and perceived social isolation of caregiving, as suggested by the (transactional and care-specific) stress-process models [5, 10]. From previous research it is known that epistemic, existential and social needs are the basis of these beliefs [20]. If higher levels of conspiracy mentality would fulfill these needs among informal caregivers during the COVID-19 pandemic, for example, by making caregivers with higher levels of conspiracy mentality feel more powerful and less uncertain, we would have expected caregivers to perceive the situation as more manageable and the outcomes to be improved. However, the opposite effect was found. This suggests that conspiracy mentality could be a risk factor for health and wellbeing of informal caregivers. This is particularly problematic during a crisis such as the COVID-19 pandemic. The pandemic already worsened the caregiving situation of informal caregivers in terms of additional care load [11, 12] and less formal support [11, 12]. COVID-19-specific caregiver stressors have also been shown to worsen psychosocial health outcomes among informal caregivers [14]. Our findings add to this, by showing that the inclination to belief in conspiracy theories is another factor that needs to be considered when looking at pandemic-specific stressors. Moreover, conspiracy mentality was a predictor of worse health and social wellbeing irrespective of the caregiving time and the social support informal caregivers reported and was associated with higher burden even if the resiliency factor of social support [55] was taken into account. Thus, believing in conspiracy theories seems to add to the worsening of health and wellbeing that has been found among informal caregivers pre- and peri-pandemic [11, 33, 56].

Additionally, our findings may also be perceived as providing support to previous findings indicating that conspiracy beliefs are not fulfilling the epistemic, existential and social motives in the general population and were therefore found to be a risk factor for psychosocial health in our study. Although further research is needed to analyze if believing in conspiracy theories can fulfill the basic needs that motivate them, there is some research already that is in line with our findings. For example, previous findings indicated that not only can feeling powerless increase conspiracy beliefs, but conspiracy beliefs also contribute to feeling powerless [37, 57]. Thus, support exists for the assumption that conspiracy theories do not fulfill the needs for safety and security, instead, they reinforce feelings of powerlessness and threat [16, 20, 37]. Among informal caregivers, conspiracy mentality also did not seem to eliminate feelings of uncertainty, threat or worries, but may instead have exacerbated them and this resulted in higher burden and worse mental health.

Further research on perceived social isolation also provides support for a negative loop between social exclusion and conspiracy beliefs. While Graeupner and Coman [58] showed that social exclusion increases conspiracy beliefs, there are various findings supporting the opposite direction of conspiracy mentality as endangering or decreasing perceived social isolation and loneliness. Believing in conspiracy theories is also associated with less normative, legal political engagement but higher willingness to engage in illegal, non-normative political actions and in general lower intentions to go along with socially accepted and desired behaviors [37, 59]. Conspiracy beliefs and mentality are also associated with more (real and hypothetical) aggressive and violent behavior [60], even against health care workers during the pandemic [61]. Additionally, they are associated with lower interpersonal trust [62], and more prejudice and stereotypic thinking towards other groups [63]. As behaviors that are non-conform with social norms and laws, conspiracy beliefs may thus promote rejection by others. This is supported by findings that believing in conspiracy theories is generally stigmatized and people are aware of this [64, 65], which can result in fear of social exclusion [38]. This fear is not unfounded. Findings from the COVID-19 pandemic indicate that higher conspiracy mentality made it more likely that others ended the contact [39]. In sum, believing in conspiracy theories does not seem to be a functional, successful strategy to achieve the fulfillment of one’s social needs and is instead more likely to be associated with more perceived social isolation and loneliness. It may instead be a maladaptive coping mechanism as has also been suggested by Heiss et al. [66]. This could explain the findings in our study, namely, why informal caregivers report more loneliness and social exclusion when reporting higher conspiracy mentality.

Further research analyzing the role of these psychological factors (i.e., the three motives) is recommended to provide insight into the underlying mechanisms of the association we found. In addition, the role of political, social, demographic and economic factors as well as further psychological factors such as perceived threat or danger by the pandemic should be analyzed as well, since they are relevant antecedents of conspiracy beliefs [16, 50, 66] and some of them also influence informal care provision [24, 67, 68]. For example, socioeconomic and demographic aspects, such as education, income, and ethnic background are associated with informal caregiving, conspiracy beliefs and health [50, 67–70]. Thus, the association between conspiracy beliefs and health may differ based on the socioeconomic status and ethnic background of the informal caregiver. It was not the aim of this study to analyze the moderating influence of these factors but further research on this would add to our findings and could help to gain a better understanding of conspiracy mentality among informal caregivers and to specify interventions and policy recommendations further.

Moreover, further dyadic research could help to analyze the effect of conspiracy beliefs among caregivers on their care recipient’s wellbeing. Previous research has already shown that burden and worse health and wellbeing of caregivers also affects care recipients [71, 72] and conspiracy beliefs may contribute to this.

### Limitations and advantages of the study

This study was a cross-sectional Online Survey therefore the causal direction could not be deduced. Longitudinal research is needed for this. Also, further pandemic indicators and socioeconomic and -demographic factors may influence the analyzed associations [16, 24, 50, 66–70]. While we could control for many of these factors, more sophisticated and validated instruments for assessing the pandemic’s impact are recommended, as well as additional research on different subgroups of informal caregivers. Further research on the post-pandemic development of the conspiracy mentality and its risk potential for wellbeing of caregivers (especially in comparison to the situation during the pandemic) could also add to our findings. Still, our study enabled insight into the caregiving situation during one of the worst phases of the COVID-19 pandemic. Also, we controlled for various context factors and characteristics to reduce bias and a large representative sample for informal caregivers aged ≥ 40 years from Germany was used. While an online bias is possible, the panel had originally been recruited via phone and the online bias is thus assumed to be negligible. Moreover, we used well-established, validated and reliable instruments for our outcomes and our main independent variable conspiracy mentality. It should also be noted that this is the first study to analyze the associations between conspiracy mentality and health and wellbeing among informal caregivers who were part of and responsible for the high risk group of the COVID-19 pandemic.

## Conclusion

This study’s findings are a first step in extending previous knowledge on informal caregiving during a societal crisis and on consequences of conspiracy mentality with a large, representative sample of informal caregivers in the second half of life (40 years and older) of older care recipients.

The findings add to the theoretical considerations of the stress-process model for informal caregivers [10], which postulate that different factors can contribute to the risk and resilience of informal caregivers regarding the consequences of informal caregiving, such as caregiving burden and wellbeing. According to our findings, conspiracy mentality seems to worsen the situation for informal caregivers during the COVID-19 pandemic, in terms of higher levels of conspiracy mentality being associated with worse mental health, higher caregiver burden and perceived social isolation and loneliness. Thus, conspiracy mentality could be one of the risk factors, which can influence this stress process among informal caregivers, irrespective of their gender.

In sum, our findings indicate that conspiracy mentality should be given more attention among particularly vulnerable subgroups of the population, such as informal caregivers, since groups in a disadvantageous position are more likely to believe in conspiracy theories [73]. Thus, conspiracy mentality should be taken into consideration in future research and in the development of supportive actions for informal caregivers. Informal caregivers could benefit, for example, from actions or campaigns aimed at reducing conspiracy mentality, since they can be expected to improve wellbeing and health in this group. These campaigns should be focusing on the underlying needs of the conspiracy beliefs (e.g., uncertainty, need for security and social connection, [20]). For example, campaigns to foster critical and analytical thinking [74] and using spokespeople who are perceived as competent and intelligent [75]. Also, placing reports on conspiracy believers into context could be helpful, by showing that conspiracy beliefs are not the norm (in their in-group) but an exception and thus provide no improved chances of social connections [76].

Moreover, reducing conspiracy mentality could also prevent further negative consequences, which have been found in the general population before and during the COVID-19 pandemic and could further worsen the situation of informal caregivers. For example, more critical health behaviors, including lower compliance with vaccination (in general) and lower likeliness to get tested during the COVID-19 pandemic [39, 77, 78], could be prevented.

## Supplementary Information

Below is the link to the electronic supplementary material.

Supplementary file1 (PDF 121 kb)
