# Supplementary material for: Conspiracy mentality among informal caregivers as a risk factor for caregiver burden, mental health, perceived loneliness and social isolation during the COVID-19 pandemic: findings of a representative online study from Germany
Source: Qual Life Res. 2022 Jul 5;31(11):3139–51. doi: 10.1007/s11136-022-03177-0 (PMC9255449; doi:10.1007/s11136-022-03177-0)
Supplement: Supplementary file 1 — Supplementary file1 (PDF 121 kb) [file 11136_2022_3177_MOESM1_ESM.pdf]

Supplementary File for the Manuscript *Zwar, König, & Hajek (2022) Conspiracy mentality among informal caregivers as a risk factor for caregiver burden, mental health, perceived loneliness and social isolation during the COVID-19 pandemic: Findings of a representative online study from Germany, Quality of Life Research.*

Appendix A: Missing values and additional analyses

Table A1 gives information on frequency and percentage of missing values in the analyzed variables of this study. Table A2 provides the results of an additional structural equation analyses using full information maximum likelihood (FIML) estimation.

Table A1
Missing values of all analyzed variables

	N (%)
Gender	0
Age	0
Highest educational degree	6 (1.23)
Marital status	2 (0.41)
Employment status	2 (0.41)
Living situation	4 (0.82)
Children in one's household	5 (1.02)
Caregiving time (h/week)	
- No information given	13 (2.66)
- Don't know	52 (10.63)
Self-rated health	0
Perceived danger for caregiver	0
Perceived danger for care recipient	
- No information given	8 (1.64)
- Don't know	1 (0.20)
Social support	
- No information given	7 (1.43)
- Don't know	11 (2.25)
Conspiracy mentality	21 (4.29)
Social exclusion	
- No information given	2 (0.41)
- Don't know	24 (4.91)
Loneliness	
- No information given	2 (0.41)
- Don't know	11 (2.25)
Burden	
- No information given	12 (2.45)
- Don't know	24 (4.91)
Depressive symptoms	
- No information given	5 (1.02)
- Don't know	21 (4.29)

Table A2

Results of structural equation models with full information maximum likelihood

	(1) Burden				(2) Loneliness				(3) Social exclusion				(4) Depressive symptoms			
	b	Robust SE	z	p	*b*	Robust SE	z	p	*b*	Robust SE	z	p	*b*	Robust SE	z	p
Conspiracy mentality	.10	.04	3.05	0.002	.01	.00	3.71	0.000	.02	.00	6.24	0.000	.10	.02	4.81	0.000
Gender (ref. male)	1.52	.78	1.95	0.052	.04	.06	0.63	0.531	.08	.06	1.36	0.173	.81	.45	1.81	0.071
Caregiving time	.05	.03	2.01	0.045	-.00	.00	-1.94	0.053	.00	.00	0.30	0.767	.00	.01	0.12	0.905
Social support	-.36	.08	-4.45	0.000	-.05	.01	-8.94	0.000	-.03	.01	-4.56	0.000	-.14	.04	-3.55	0.000
Age	-.05	.05	-0.93	0.352	-.00	.00	-1.11	0.265	-.01	.00	-1.70	0.089	-.14	.04	-3.94	0.000
Marital status (ref. Married)																
- divorced	-1.58	1.24	-1.27	0.202	-.03	.08	-0.35	0.723	.11	.10	1.14	0.254	-.13	.70	-0.18	0.856
- widowed	-2.71	2.17	-1.25	.212	.26	.15	1.77	0.076	.02	.09	0.28	0.781	1.18	.82	1.44	0.149
- single	-2.15	1.17	-1.84	.066	.13	.11	1.23	0.219	.23	.10	2.24	0.025	-.28	.78	-0.35	0.725
Highest educational degree (ref. upper secondary school)																
- lower secondary school	-.91	1.06	-.85	.393	-.03	.09	-0.31	0.756	.01	.08	0.16	0.872	-1.01	.61	-1.67	0.095
- intermediate secondary school	1.05	.92	1.14	.252	-.02	.07	-0.24	0.814	.09	.07	1.33	0.185	-.09	.53	-0.17	0.866
- polytechnic secondary school	-.23	1.48	-.16	.874	-.06	.11	-0.57	0.565	-.01	.09	-0.06	0.955	-1.01	.78	-1.30	0.195
- qualification for applied	.14	1.28	0.11	.913	.17	.10	1.70	0.090	.15	.10	1.51	0.131	.29	.76	0.38	0.703

upper secondary school																
Employment status (ref. employed (full-time))																
- employed (part-time)	-.51	.99	-0.51	0.607	-.00	.08	-0.03	0.972	-.02	.07	-0.24	0.811	-.73	.50	-1.45	0.148
- marginally employed	-4.07	1.79	-2.27	0.023	-.14	.14	-1.00	0.315	-.25	.13	-1.98	0.048	-.97	.72	-1.34	0.181
- retired	-1.16	1.19	-0.97	0.330	-.18	.09	-1.99	0.046	-.03	.09	-0.33	0.740	.18	.70	0.25	0.800
- unemployed	-.64	1.54	-0.42	0.677	.01	.10	0.06	0.953	.32	.12	2.68	0.007	1.44	.86	1.67	0.094
Living situation (ref. living alone in private household)																
- living together with other ins private household	-1.14	1.20	-0.95	0.342	-.01	.09	-0.10	0.916	-.06	.08	-0.75	0.452	.19	.65	0.30	0.763
- living in assisted living/nursing care home/retirement home	-6.09	2.32	-2.62	0.009	-.27	.13	-2.04	0.042	-.60	.23	-2.67	0.008	-1.00	1.42	-0.71	0.479
Children in one’s household (ref. None)																
- Yes, younger than 14 years	-2.01	1.20	-1.68	0.093	.02	.10	0.15	0.881	.19	.12	1.63	0.104	-1.02	.69	-1.49	0.137
- Yes, between 14 and 18 years	.55	1.08	0.51	0.613	-.01	.09	-0.07	0.947	-.06	.07	-0.90	0.369	-.06	.66	-0.09	0.932
Self-rated health	-1.34	.47	-2.89	0.004	-.15	.03	-4.83	0.000	-.16	.03	-4.83	0.000	-1.89	.27	-7.05	0.000

Perceived danger for caregiver (oneself)	.76	.42	1.82	0.069	.05	.03	1.70	0.089	.08	.03	2.45	0.014	.22	.26	0.84	0.400
Perceived danger for care recipient	.18	.32	0.56	0.576	.00	.03	0.06	0.954	.05	.03	1.97	0.049	.41	.20	2.04	0.042
Constant	17.46	4.57	3.82	0.000	3.28	.33	9.95	0.000	2.04	.34	5.95	0.000	18.23	2.72	6.69	0.000
Observations	387				398				387				389			

Note. Structural equation analyses using full informal maximum likelihood estimation were conducted; unstandardized regression coefficients and robust standard errors are given. *Conspiracy mentality* (Range: 0-50), higher scores indicate higher conspiracy mentality; *burden* (BSFCs), Range: 0-30, higher scores indicate higher caregiver burden; *loneliness* (de Jong Gierveld Scale) Range: 1-4, higher scores indicate higher loneliness; *social exclusion* (Bude & Lantermann) scale Range: 1-4, higher scores indicate higher social exclusion; *depressive symptoms* (PHQ-9, Range: 0-27), higher scores indicate more depressive symptoms; *self-rated health* (Range 1-5), higher scores indicate better health; *social support* (Lubben's social network scale) Range: 0-30 (family and network scale Range: 0-15), higher scores indicate stronger social network and more social support; *perceived danger for oneself* (caregiver) *and for care recipient* (Range: 0-5), higher scores indicate higher levels of perceived danger Level of significance: *** p<0.001, ** p<0.01, * p<0.05, + p<0.10
